# Influence of shot peening on the microstructure and friction-wear performance of CF53 steel

**DOI:** 10.1371/journal.pone.0317410

**Published:** 2025-06-02

**Authors:** Qiushen Cai, Huashen Guan, Junjie Zhang, Xiaoguang Zhang, Chenfeng Duan

**Affiliations:** 1 Jiangmen Power Supply Bureau of Guangdong Power Grid Co. Ltd., Jiangmen, Guangdong, China; 2 School of Mechanical and Automotive Engineering, South China University of Technology, Guangzhou, China; Himachal Pradesh University, INDIA

## Abstract

In order to further enhance the wear resistance of the camshaft surface, this study conducted shot peening reinforcement on CF53 steel. The research involves the analysis of microstructure, microhardness, and residual stress evolution. Additionally, a pin-on-disk friction and wear test machine was used to investigate the influence of shot peening on the friction-wear characteristics of CF53 steel. The results show that a certain depth of plastic deformation layer is formed on the surface after shot peening, accompanied by an increase in surface roughness. With the increase of shot peening pressure, the surface roughness, microhardness, and residual stress values of the samples correspondingly increase. Shot peening treatment significantly improves the friction-wear performance of CF53 steel, with a reduction of 22.5% and 54.6% in the friction coefficient and wear rate for SP3 and SP4 groups, respectively, compared to untreated samples. The wear mechanism of untreated samples is characterized by severe fatigue wear with prominent features of plowing grooves and cracks. In contrast, the wear mechanism of peened samples shifts to fatigue wear dominated by delamination.

## 1. Introduction

The camshaft is one of the five key components of a diesel engine, responsible for controlling the opening and closing of valves. During operation, the camshaft experiences bending and torsional loads. This is because the force exerted by the valve spring during the rotation of the camshaft also causes bending loads on the camshaft. In addition, during the power transmission process, the camshaft is subjected to torque due to factors such as friction and inertia in the transmission system. Therefore, the cam portions endure varying compressive stresses along with friction from the tappets. Consequently, the surface is prone to wear, which can adversely affect the efficiency and lifespan of the diesel engine. CF53 steel, characterized by high strength, carbon structure, good hardenability, and machinability, finds extensive applications in the manufacturing of crucial components such as crankshafts, machine tool spindles, camshafts, and rollers [1]. However, similar to many other medium-carbon steels, CF53 steel exhibits notable shortcomings in terms of wear resistance [2].

Shot peening is a surface treatment method that introduces residual stresses and induces work hardening on the material surface. In comparison with other strengthening processes, its direct advantages lie in simple equipment, low processing costs, and wide applications in the fields of power machinery and aerospace [3,4]. Through the repetitive impact of high-speed projectiles on the workpiece surface, shot peening induces the formation of a stable elasto-plastic deformation layer on the surface. This uneven deformation introduces a residual compressive stress layer in the material surface [5], Simultaneously, the microstructure within the deformed layer undergoes changes after shot peening, with a tendency towards nanoscale grains. The arrangement of grains becomes denser, and grain refinement contributes to enhancing the surface hardness of the material [6]. Currently, research on shot peening for improving the friction-wear performance of materials has been reported. Yang et al. [7] conducted experimental studies on the micro-wear behavior of Ti-6Al-4V after shot peening, revealing an optimal combination of hardness and toughness on the surface material after moderate intensity (0.3 mmA) shot peening. This effectively reduced the wear rate of Ti-6Al-4V during prolonged micro-wear processes. Kumar et al. [8] found that the wear rate of medium-carbon steel (AISI 6150 steel) decreased by 20–30% after shot peening, but further increases in shot peening intensity did not lead to any significant improvement in wear resistance. Zhang et al. [9] conducted pin-on-disk friction tests on shot-peened 17Cr2Ni2MoVNb steel and found that SP-treated samples exhibited lower friction coefficients and better wear resistance. Gopi et al. [10] observed that shot peening could improve the surface and friction performance of 316L stainless steel, noting higher dislocation density and lateral grain elongation on the shot-peened sample surface. Additionally, shot peening significantly increased surface hardness without altering the metal phase composition.

Currently, there is limited research on shot peening reinforcement for CF53 steel, and the understanding of the patterns that shot peening influences its surface quality and subsequently alters friction-wear performance is not well-established. Therefore, the purpose of this study is to investigate the evolution patterns of microstructure, phases, hardness, and residual stress in CF53 steel after shot peening treatment. Additionally, sliding wear tests will be conducted to assess the wear resistance of CF53 steel after shot peening reinforcement. The study aims to unveil the changes in the material’s tribological behavior after shot peening through the analysis of friction-wear test results and morphology. Furthermore, an analysis of the corresponding surface wear mechanisms will be conducted. This research intends to provide essential technical references for enhancing the surface wear resistance of CF53 steel in engineering applications.

## 2. Experimental materials and methods

### 2.1. Material preparation

The CF53 steel used in the experiment was provided by Weichai Power Co., Ltd. The chemical composition (wt.%) is as follows: 0.613 C, 0.184 Si, 0.667 Mn, 0.136 Cr, 0.036 Ni, 0.013 Al, 0.017 P, 0.005 S, and the balance Fe. Before shot peening treatment, the test steel underwent a heat treatment process, with a 25-minute soaking at 800 °C, followed by quenching in a 15% NaCl solution. Subsequently, the samples were kept at 360 °C for 2 hours and then air-cooled to room temperature. After heat treatment, the microhardness of the test steel was measured to be 481.2 HV, the tensile strength was 1580 MPa, and the elongation at fracture was 9.3%.

### 2.2. Shot peening

Shot peening was performed using an LSWPC1010FK-A pneumatic shot peening machine. Prior to shot peening, disk-shaped samples with dimensions of φ43 mm × 4 mm were cut from the heat-treated test steel. The surface oxide layer was removed, and the samples were polished and cleaned with anhydrous ethanol using ultrasonication. LSGH110 commercial alloy steel shots with an average diameter of 0.6 mm were used for shot peening, achieving a coverage rate of 200%. The shot peening intensity was determined by the arc height of A-type Almen strips. The shot peening height was set at 140 mm, and the spraying angle was 90°. The shot peening pressures for SP1-SP5 are 0.2MPa, 0.3MPa, 0.4MPa, 0.5MPa, and 0.6MPa, respectively. The intensity after spraying is 0.266mmA, 0.345mmA, 0.379mmA, 0.425mmA, and 0.470mmA, respectively.

### 2.3. Wear test

Sliding wear tests were conducted at room temperature (25 °C) using an MMU-10G end-face friction and wear testing machine with a pin-on-disk contact configuration. The upper specimen was a GCr15 steel ball with a diameter of 3 mm and a hardness of 675.0 HV_0.2_. The lower specimen was a disk-shaped CF53 steel sample. Each test was repeated three times. The tests were performed under the conditions of a spindle speed of 60 r/min, a load of 300 N, and a test duration of 30 minutes. The lubricating oil used in the tests was Mobil SpectraSynTM 10 synthetic base oil. This choice aimed to avoid potential influences from additives in commercial lubricants on the wear results. The lubricating oil had a viscosity index of 137, a kinematic viscosity of 66 cSt at 40°C, and a kinematic viscosity of 10.0 cSt at 110°C.

After the completion of the friction-wear tests, measurements of the cross-sectional profiles were taken at six points along each wear scar. The maximum and minimum values were excluded, and the average depth of the wear scar (*h*) was then determined. The wear volume (*V*) and volume wear rate (*K*) were calculated using the following formulas [11]:


$$V=\left(r^{2}\arccos\left(\frac{r-h}{r}\right)-(r-hsqrt2rh-h^{2}\right)\times l$$ (1)

where, l is the length of the annular wear scar, r is the radius of the spherical pin, and h is the average depth of the wear scar.


$$K=\frac{V}{Fs}$$ (2)

where, *V* represents the wear volume (m³), *F* is the friction load (N), and *s* is the total relative wear travel of the spherical pin with respect to the lower specimen.

### 2.4. Measurement and analysis methods

**Fig 1:**
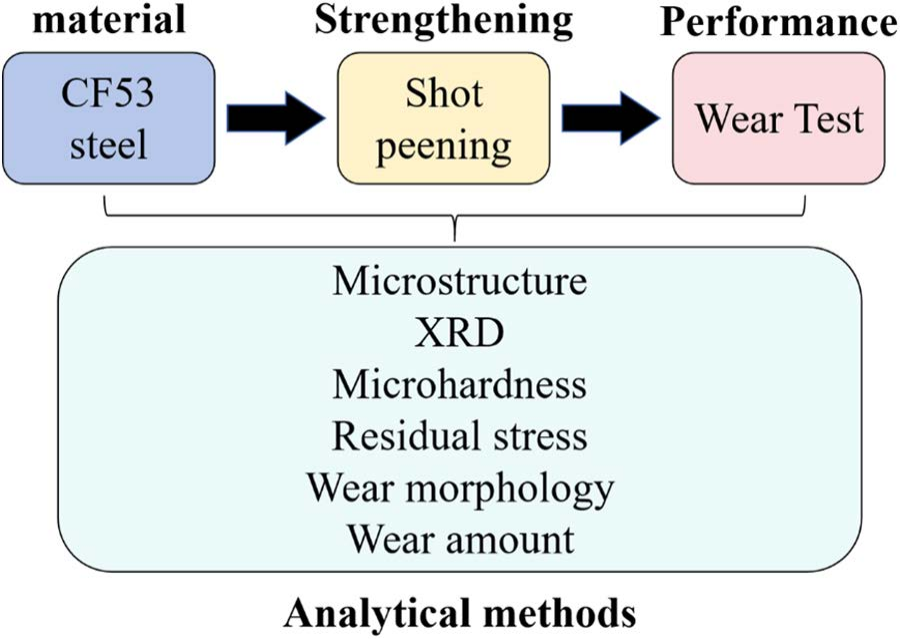
Testing schedule.

The surface roughness of the samples was measured using a MARSURF-M300C roughness tester. The cross-sectional hardness of the samples was measured using an SCTMC-HV50 micro Vickers hardness tester with a test load of 200 g and a loading time of 15 s. After corrosion polishing the sample surfaces with a 4% nitric acid alcohol solution, the microstructure of the cross-section was observed using a LEICA-M165C optical microscope. The surface phase composition of the samples was examined using a Bruker D8-Advance X-ray diffractometer. Surface residual stresses were measured using a Proto-LXRD X-ray stress analyzer. The surface morphology and wear profiles of the samples were observed using a RTEC UP Dual-Mode 3D optical profiler. The surface morphologies before and after shot peening, as well as before and after wear, were analyzed using a Quanta 200 environmental scanning electron microscope (SEM) combined with an energy-dispersive X-ray spectrometer (EDS). The overall testing schedule used in this paper is shown in Fig 1.

## 3. Results and discussion

### 3.1. Surface morphology and roughness

**Fig 2:**
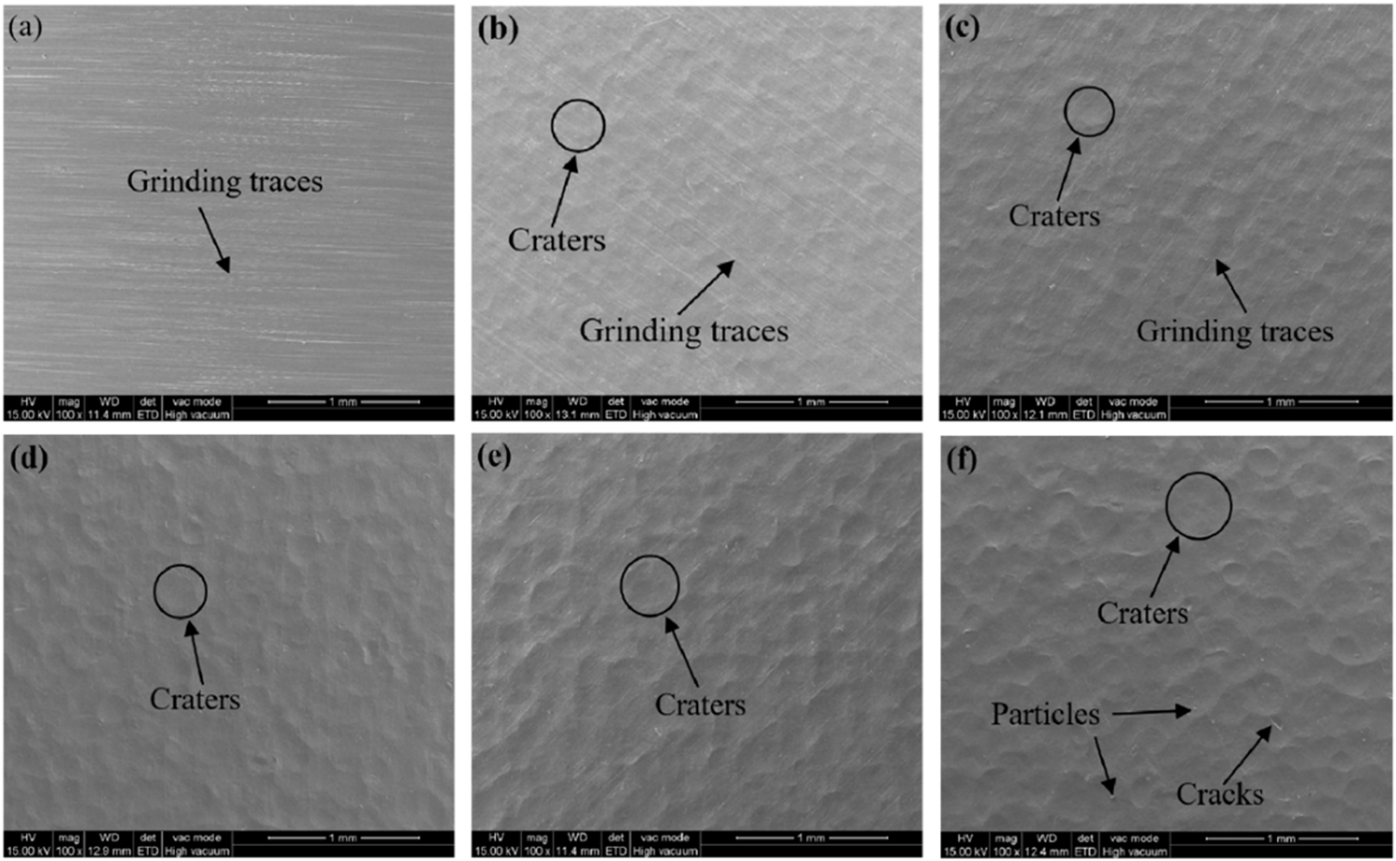
Surface morphology of various samples (a) Untreated, (b) SP1, (c) SP2, (d) SP3, (e) SP4, (f) SP5.

Fig 2 presents the SEM images of the sample surfaces before and after shot peening. From Fig 2a, it can be observed that the untreated sample surface exhibits evenly distributed fine grinding scratches. As the shot peening pressure increases, the grinding scratches on the sample surface gradually disappear, and various-sized dimples appear. The surfaces of the SP1 and SP2 samples still show a small amount of grinding scratches, with shallow dimple traces. When the shot peening pressure reaches 0.4 MPa, the grinding scratches on the surfaces of the SP3, SP4, and SP5 samples are virtually eliminated, and the dimple traces become more noticeable and larger. However, the surface of the SP5 sample exhibits debris and microcracks, suggesting that intense plastic deformation may lead to surface defects, thereby affecting the surface quality of this sample. The surface roughness (Ra) of the untreated sample is 0.627 μm. The Ra values for the SP1, SP2, SP3, SP4, and SP5 samples are 1.659 μm, 1.994 μm, 2.192 μm, 2.309 μm, and 2.569 μm, respectively. Compared to the untreated sample, these values represent increases of 164.6%, 218.0%, 249.6%, 268.3%, and 309.7%, respectively. With the increase in shot peening pressure, the surface roughness of the samples also increases. This is mainly attributed to the higher initial velocity of the projectiles with greater shot peening pressure, leading to more significant material surface plastic flow and increased surface undulation, ultimately resulting in higher surface roughness.

### 3.2. Microstructure and phase analysis

**Fig 3:**
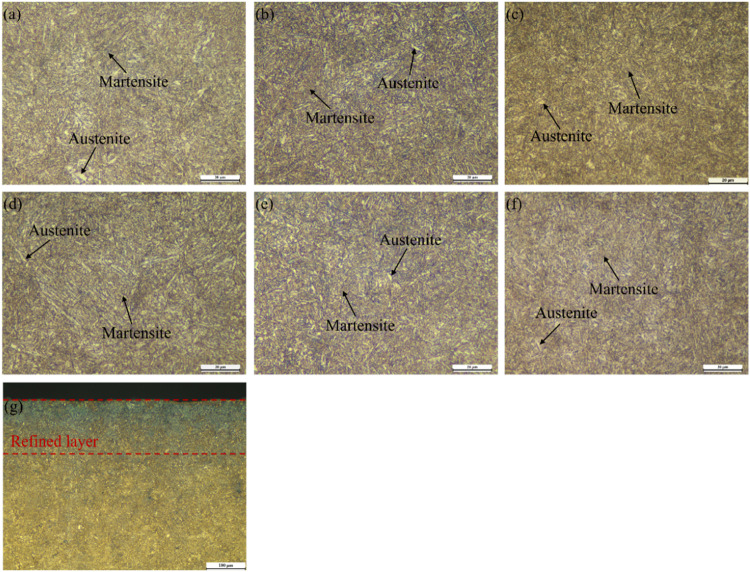
Microstructures of various samples (a) Untreated, (b) SP1, (c) SP2, (d) SP3, (e) SP4, (f) SP5, (6) Cross-section of SP5 specimen.

The surface microstructures of the samples are shown in Fig 3a–f. Both before and after shot peening, the surface microstructure of the samples consists mainly of lath-shaped and needle-shaped martensite mixtures as well as residual austenite. It can be observed that shot peening did not alter the material’s structural composition, but the surface microstructure was significantly refined, and the martensite structure gradually increased. Fig 3g displays the cross-sectional microstructure of the SP5 sample, revealing the formation of a refined structure layer within a certain depth after shot peening. Closer to the sample’s core, the microstructure becomes more similar to that of the untreated sample. During shot peening, the collision of projectiles with the sample surface induces compressive deformation, accompanied by repeated crystal slip and an increase in dislocation density. The surface dislocations adapt to plastic strain through accumulation and rearrangement.

**Fig 4:**
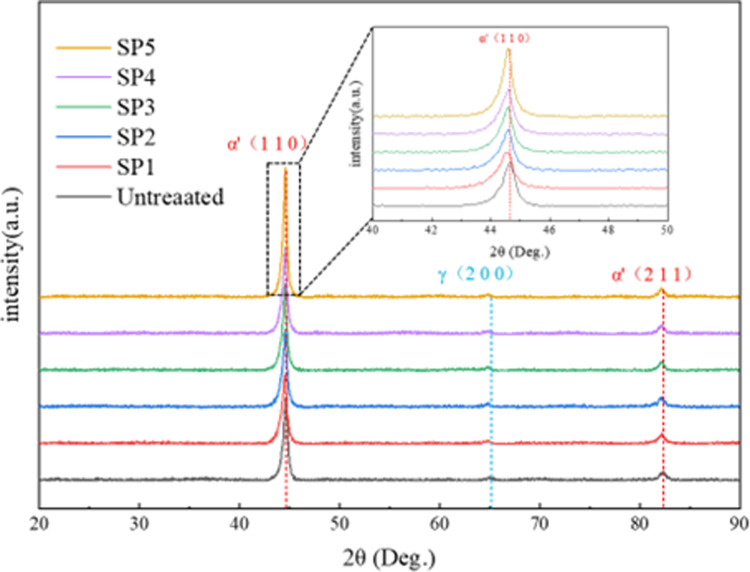
XRD of various samples.

X-ray diffraction patterns of the sample surfaces before and after shot peening are shown in Fig 4. The surface of the untreated sample is primarily composed of Fe phases. After shot peening at different pressures, the surface phase composition remains unchanged, still consisting of Fe phases. However, the diffraction peaks shift to the left and broaden. This is mainly attributed to strain deformation in the material surface, increased dislocation density, and changes in grain size [12]. As the shot peening pressure increases, the degree of peak broadening also increases. At higher shot peening pressures, the impact energy of the projectiles on the sample is greater, leading to severe plastic deformation of the surface grains, lattice distortion, and more pronounced peak broadening.

### 3.3. Microhardness

**Fig 5:**
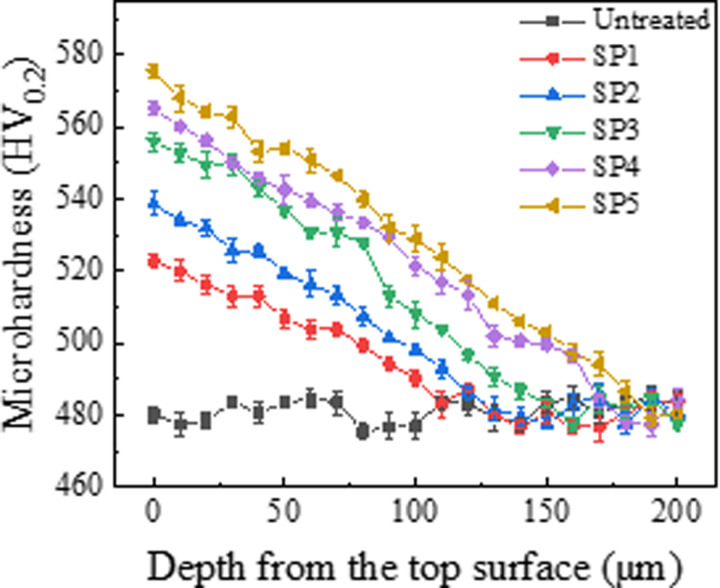
Microhardness of various samples.

The variation in microhardness with depth on the cross-sections of CF53 steel samples before and after shot peening at different pressures is shown in Fig 5. The microhardness of the untreated sample is approximately 481.2 HV0.2. After shot peening, the surface microhardness of the samples increases. The influence of shot peening decreases with increasing distance from the surface, gradually approaching the microhardness of the matrix. This indicates the formation of a hardened zone with a certain depth on the surface of the samples. The surface hardness of the SP1, SP2, SP3, SP4, and SP5 samples is 522.5 HV_0.2_, 538.5 HV_0.2_, 555.9 HV_0.2_, 565.2 HV_0.2_, and 575.5 HV_0.2_, respectively, representing increases of 8.6%, 11.9%, 15.5%, 17.5%, and 19.6% compared to the untreated sample. Research by Hassani et al. [13] suggests that the increase in material microhardness is primarily due to the refinement of surface grains and plastic deformation within the strengthening layer. Higher shot peening pressures give projectiles higher initial velocities, resulting in higher kinetic energy that leads to severe plastic deformation in the surface layer of the sample. This process produces a dense microstructure, making dislocation movement difficult, and manifests as significant work hardening. As a result, the SP5 sample has a higher microhardness than the other four groups.

### 3.4. Residual stress

**Fig 6:**
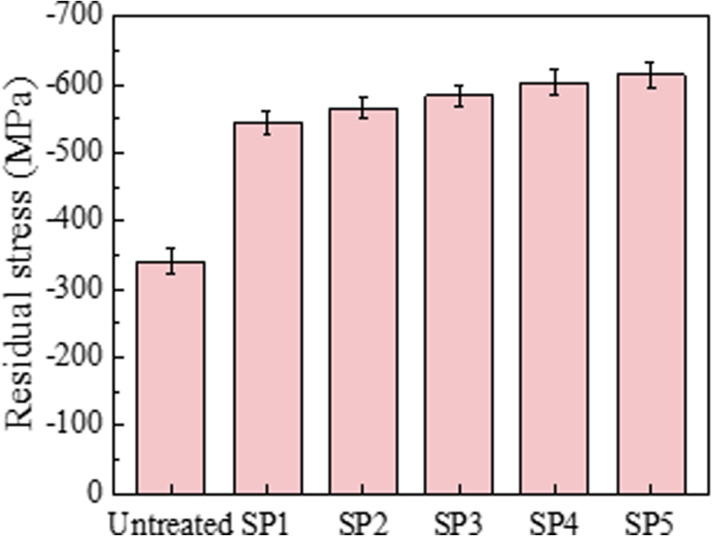
Residual stress of various samples.

Fig 6 shows the surface residual stress of CF53 steel samples. Residual compressive stress was detected on the surface of all samples. Due to the influence of heat treatment and mechanical processing, the untreated sample’s surface also introduced a residual compressive stress of -340 MPa. As the shot peening pressure increases, the introduced residual compressive stress on the surface of the SP samples gradually increases. The surface residual stresses of the SP1, SP2, SP3, SP4, and SP5 samples are -544 MPa, -566 MPa, -584 MPa, -603 MPa, and -615 MPa, respectively, representing increases of 60.0%, 66.5%, 71.8%, 77.4%, and 80.9% compared to the untreated sample. During the shot peening process, most of the projectile’s energy is converted into elastic potential energy and plastic deformation energy in the deformation layer. Higher shot peening pressures lead to severe plastic deformation and an increased degree of uneven plastic deformation. Therefore, higher shot peening pressures increase the residual compressive stress values introduced on the sample’s surface.

### 3.5. Friction and wear behavior

#### 3.5.1. Effect of shot peening on material friction characteristics.

**Fig 7:**
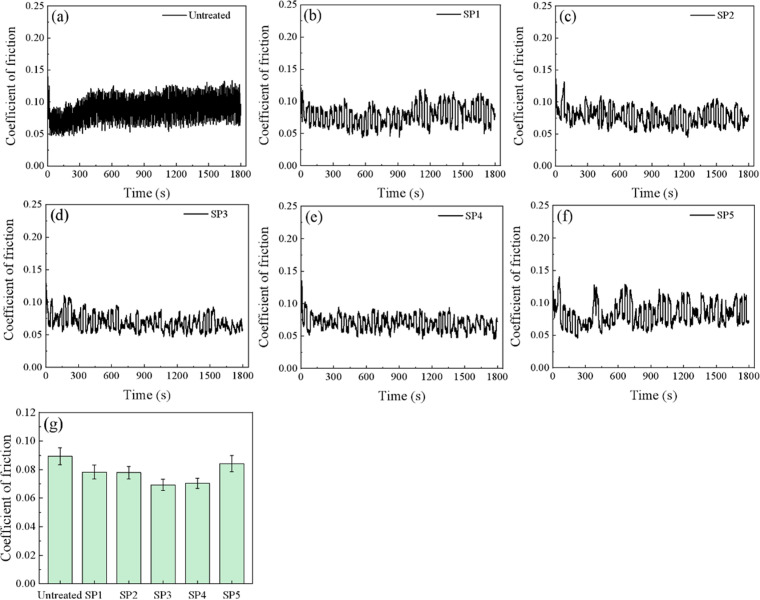
Friction coefficients of various types of specimens (a) Untreated, (b) SP1, (c) SP2, (d) SP3, (e) SP4, (f) SP5, (6) Average friction coefficient.

Fig 7 shows the friction coefficient curves of CF53 steel samples with and without shot peening treatment. In the initial stage of wear, the rough peaks in contact deform or even wear off, causing a sharp increase in the friction coefficient curve of the untreated sample. After about 420 s, it enters a stable wear stage, during which the surface friction coefficient fluctuates significantly and remains within the range of 0.06 to 0.12 until the end of the test. The friction coefficient curves of the SP samples, as shown in Fig 7b–f, indicate a significant reduction in the fluctuation range, and the overall friction coefficient curves roughly follow a linear distribution. The friction coefficient of the SP3 sample rapidly decreases after about 220 s and fluctuates within the range of 0.04 to 0.09. The friction coefficient of the SP4 sample fluctuates within the range of 0.05 to 0.08 after about 85 s. As the shot peening intensity increases further, the fluctuation range of the friction coefficient in the SP5 sample does not decrease further. Instead, it rapidly rises to around 0.1 after about 400 s, showing an overall upward trend in the subsequent wear process. Fig 7g shows the average friction coefficients of each sample. It can be observed that the untreated sample has the highest average friction coefficient, reaching 0.0894, while the shot peened samples exhibit lower average friction coefficients. The average friction coefficients of SP1, SP2, SP3, SP4, and SP5 samples are 0.0783, 0.0779, 0.0693, 0.0704, and 0.0841, respectively. With the increase in shot peening intensity, the average friction coefficient of the samples shows a trend of initially decreasing and then increasing. On the one hand, the hardening effect of the hardened zone formed by shot peening is beneficial for reducing the friction coefficient. On the other hand, the hard debris particles formed by the worn-off rough peaks may temporarily stay in the contact zone, not easily crushed or spilled, and participate in the friction between the mating surfaces. At this point, the friction pair changes from two-body wear to three-body wear. Diomidis et al. [14] pointed out that the third body formed by the wear debris would affect the displacement adaptability in the contact zone, thus affecting the friction coefficient. At the same time, it can also separate the contacting surfaces, providing a certain protective effect.

#### 3.5.2. Impact of shot peening on material wear characteristics.

**Fig 8:**
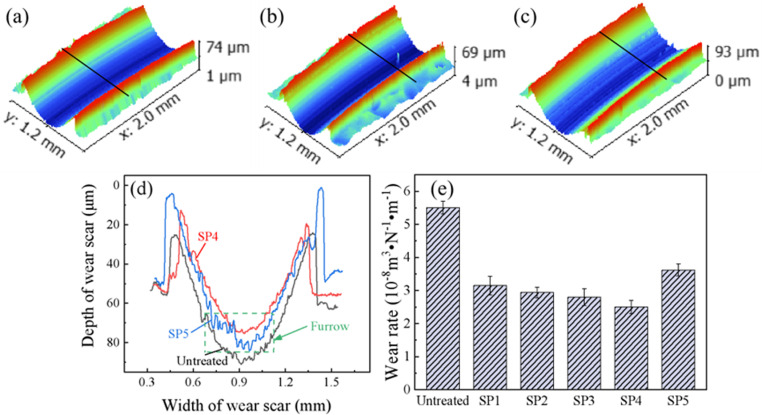
Wear profiles and wear rates of various types of specimens (a) Untreated, (b) SP4, (c) SP5, (d) Wear profiles, (e) Wear rates.

Fig 8 shows the wear profiles and wear rate calculation results of CF53 steel samples with different shot peening intensities before and after sliding wear tests. The wear depth of shot peened samples is significantly reduced, but the morphology at the bottom of the wear scar becomes rugged, and even sharp grooves appear at higher shot peening pressures. It is speculated that with the increase in shot peening pressure, the surface roughness increases, and the hardening effect of the surface cold work strengthens. The protrusions left by shot peening may generate hard cutting debris during the wear process, while the pits play a role in storing oil lubrication. The two compete in the friction pair, eventually forming a bottom surface morphology similar to that of the SP5 sample. Combining the wear rate calculation results in Fig 8e, shot peened samples exhibit a significantly reduced wear rate. The wear rate of the untreated sample is 5.51 × 10^-8^ m^3^·N^-1^·m^-1^, while the wear rates of SP1, SP2, SP3, SP4, and SP5 samples decrease to 3.15 × 10^-8^ m^3^·N^-1^·m^-1^, 2.94 × 10^-8^ m^3^·N^-1^·m^-1^, 2.80 × 10^-8^ m^3^·N^-1^·m^-1^, 2.50 × 10^-8^ m^3^·N^-1^·m^-1^, and 3.62 × 10^-8^ m^3^·N^-1^·m^-1^, respectively. It is evident that the wear rate of the SP4 sample has the most significant reduction, reaching 54.6%. Notably, with the increase in shot peening pressure, the wear rate of the samples shows a trend of initially decreasing and then increasing. The study by Han et al. [15] indicates that the introduction of a residual stress field on the surface of the sample after shot peening can effectively resist some stress during sliding wear, thus slowing down wear. In addition, the craters on the surface of shot peened samples have oil storage and lubrication functions during wear, which can increase the oil film thickness and effectively isolate the contact before the friction pair, contributing positively to the reduction of the wear rate.

#### 3.5.3. Surface wear morphology and wear mechanism analysis.

**Fig 9:**
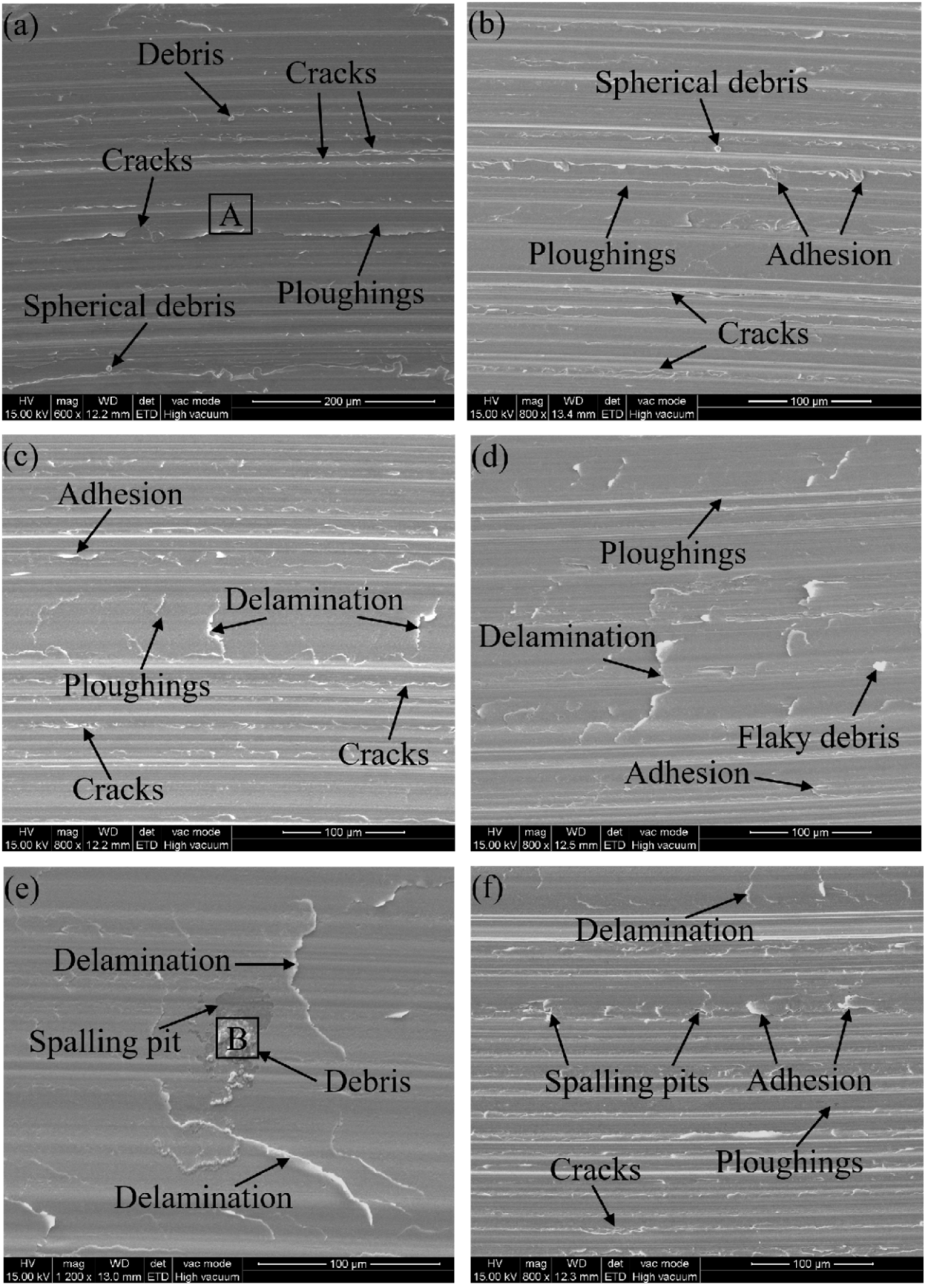
Wear morphology of various types of specimens (a) Untreated, (b) SP1, (c) SP2, (d) SP3, (e) SP4, (f) SP5.

The surface wear morphology of CF53 steel samples before and after shot peening at different pressures is shown in Fig 9. Observing Fig 9a, it is found that there are microcracks at the edges of the wear surface furrows, accompanied by a considerable amount of wear debris. Due to the lower surface roughness and hardness of the untreated sample and weaker load-bearing capacity, the sample surface is difficult to form a stable and sufficiently thick lubricating oil film during sliding wear. The surface material is prone to shear spalling under the cutting stress of the ball head pin. With the increase in shot peening pressure, the characteristics of shear deformation-induced spalling become more apparent. It is worth noting that the wear morphology of fatigue mechanisms during sliding wear often exhibits spalling and wear debris [16]. Fig 9b shows that the wear surface of the SP1 sample has spherical wear debris, small bonded flakes, and short microcracks between the furrows. Observing Fig 9c, when the shot peening pressure is increased to 0.3 MPa, the wear surface of the shot-peened sample shows layering marks perpendicular to the sliding direction, with reduced material bonding and short microcracks still present between the furrows. Compared to the sample shot peened at 0.2 MPa, the surface hardness of this sample further increases, which is conducive to improving the material’s anti-adhesion performance. Therefore, the material bonding is reduced, and the wear resistance is enhanced. However, similar to the surface of the sample shot peened at 0.2 MPa, there are still machining defects not covered by craters, leading to the initiation of a small number of microcracks.

Observing Fig 9d, the layering marks on the wear surface of the sample shot peened at 0.4 MPa become longer and more numerous, with local traces of metal plastic flow and wear debris, but no cracks are observed. The machining marks on the surface of the sample shot peened at 0.4 MPa are mostly covered by craters, and significant residual compressive stress is introduced, which is beneficial for suppressing the initiation and propagation of fatigue cracks. When the shot peening pressure is increased to 0.5 MPa, as seen in Fig 9e, the layering phenomenon becomes more pronounced, the furrow marks lighten, and large pieces of wear debris and spalling pits appear. The middle part of the wear track becomes smoother, and the friction coefficient curve of this sample also roughly shows a linear distribution, indicating a relatively stable wear process. At this point, a deeper hardened layer is formed on the surface of the sample, enhancing its load-bearing capacity. The wear mechanism is similar to that of the sample shot peened at 0.4 MPa, but due to the larger and deeper craters on its surface, the elongated layering marks formed after shear deformation of the material are more narrow. Observing Fig 9f, after shot peening at 0.6 MPa, the wear morphology of the sample is not improved with increasing pressure. The layering marks lighten, and spalling pits and microcracks appear. When using a higher shot peening pressure, firstly, the increase in surface roughness may lead to the formation of more wear debris during the friction process, and these wear debris, with higher hardness than the sample surface, are not easy to embed into the surface, resulting in “third-body” participation in the wear between the friction pairs, forming metal micro-cutting on the friction pair surface, leading to deepening furrows. Secondly, due to the extremely high contact stress between the abrasive particles in three-body abrasion and the metal surface, it is more likely to cause plastic deformation and spalling of the metal surface. Finally, the sample may experience “over-peening,” where a small number of microcracks are formed on the surface after shot peening. In the subsequent friction process, the continuously ground wear debris may press and extend the surface cracks, leading to material spalling.

## 4. Conclusion

(1) Shot peening significantly refines the surface microstructure of CF53 steel specimens. The refinement degree gradually decreases from the surface towards the core, ultimately transitioning to the base matrix. With the increase in shot peening pressure, the surface roughness, microhardness values, and residual stress values of the specimens also increase.
(2) Shot peening effectively reduces the average friction coefficient of CF53 steel specimens. As the shot peening pressure increases, the surface roughness of the specimens increases, and the average friction coefficient exhibits a “V” shaped trend with an initial decrease followed by an increase. After shot peening, the average friction coefficients of SP1, SP2, SP3, SP4, and SP5 specimens are 0.0783, 0.0779, 0.0693, 0.0704, and 0.0841, respectively, representing reductions of 12.4%, 12.8%, 22.5%, 21.3%, and 5.9%, compared to the untreated specimen with a coefficient of 0.0894.
(3) Shot peening significantly reduces the wear rate of CF53 steel specimens, enhancing the material’s anti-friction and wear-resistant properties. Among them, the wear rate of the SP4 specimen (2.50 × 10–8 m3·N-1·m-1) shows the most significant reduction, with a decrease of 54.6% compared to the untreated specimen’s wear rate of 5.51 × 10–8 m3·N-1·m-1. The wear mechanism of the untreated specimen is characterized by severe fatigue wear with prominent furrows and cracks. After shot peening, the wear mechanism transitions to fatigue wear dominated by delamination features.

### Nomenclature

**Table:** 

SP	Shot peening
h	wear scar
V	wear volume
K	volume wear rate
F	friction load
s	total relative wear travel
